# Optimized routing with Ant Colony Algorithms to extend network lifetime in Wireless Sensor Networks

**DOI:** 10.1038/s41598-026-53451-1

**Published:** 2026-05-24

**Authors:** R. Kandasamy, S. Anbu Karuppusamy

**Affiliations:** Department of Electronics & Communication Engineering, Excel Engineering College, Namakkal, 637303 India

**Keywords:** Wireless Sensor Networks, Ant Colony Optimization, Energy-aware routing, Network lifetime, MANET, Energy science and technology, Engineering, Mathematics and computing

## Abstract

Energy-efficient routing in Wireless Sensor Networks (WSNs) is a critical challenge due to uneven energy depletion and dynamic topology changes. The paper suggests a Lifetime-Aware Ant Colony Optimization-based Routing Algorithm (LTAWSN) which incorporates the residual energy, hop count and spatial proximity to probability routing. The proposed approach, in contrast to the traditional Ant Colony Optimization (ACA) and Energy-Aware ACA (EAACA) uses two energy metrics and spatial awareness to balance energy usage and enhance routing efficiency. LTAWSN performance is measured by using NS-2 simulations and compared to the performance of ACLR and ACA and EAACA. The simulation outcomes indicate that LTAWSN can save the energy consumption by 18–25%, enhancement in the percentage of packet delivery (PDR) by 6–10%, and network lifetime in different node densities. These findings verify the suitability of the proposed strategy to increase the network stability, reliability, and energy balancing in WSNs settings.

## Introduction

Ad-hoc networks are the spontaneous multi-hop wireless networks that develop without the requirement of any infrastructure. They are also able to be sent to any location even without the use of permanent facilities such as base stations. In such networks, nodes link up and automatically organize themselves into an effective network and work together to preserve network connectivity. In cases whereby two nodes willing to communicate are not in immediate broadcasting distance, they use a mediator node to pass packets to one another. As a result, the nodes accomplish two roles, which are being a router and a host. They exchange control packets that give information about the network topology and information permitting them to find multi-hop paths into which they can get connected with each other. Ad-hoc networks are usually used in scenarios when there is either a lack or insufficiency of infrastructure and the situations when the speed of network deployment and self-configuration is required. As an illustration, soldiers, in battlefield situations, can communicate with each other by the use of cell phones. Similarly, in case of emergencies such as an earthquake, where any of the current infrastructures may be destroyed, an ad hoc network can be created very quickly to support disaster response operations. Another example of ad hoc networks is sensor systems, where sensors are capable of collecting various data and communicating with one another.

In the past few years, there has been a significant focus on ad hoc network routing^1–6^. Conventional routing protocols can be categorized into three types: source-driven (reactive/on-demand), database-driven (proactive), and hybrid. In source-driven protocols, routes are not calculated until they are needed, which can result in delays in route computation^7^. In situations where such delays are unacceptable, table-driven protocols are used. These protocols pre-compute routes to all destinations and store them as routing information. Nodes in the network regularly exchange routing table update packets to maintain this information.

Routing protocols in their early development primarily emphasized the task of maintaining network connectivity within highly dynamic environments^8^.These protocols primarily dealt with issues such as route discovery and management. MANET, which stands for Mobile Ad-Hoc Network, represents a self-contained network comprised of mobile nodes and their associated hosts connected via wireless communication. In MANET, there is no permanent infrastructure or centralized control, and mobile nodes establish connections in an arbitrary manner. The networks are generally categorized into two types based on their infrastructure^9^. The initial category comprises networks incorporating base stations, whereas the second category, Mobile Ad-Hoc Networks, allows communication between devices without relying on any fixed infrastructure and is not constrained by geographical regions^10^.

The aim of this research is to tackle the challenge of enhancing the security and energy efficiency of transportation to extend the network’s longevity^11^. A mobile Ad-Hoc network is characterized by a topology of the network changing randomly at random events, which happens when the node moves randomly^12^. The network topology may also be affected by transmission and reception criteria. As a result, the prevention and maintenance of optimal power-efficient path becomes a complicated issue. Confidentiality, identification, authentication, non-repudiation, reliability and access controls are some basic security requirements of any system.

Encryption of information as well as management packets is one way that secrecy is maintained and the eavesdroppers will be unable to understand information in the network. The verification protects nodes that are adjacent to impersonation and authenticates their identities^13^. Packet integrity will ensure that packets are still unmodified and are not compromised with by any malicious users. Wireless sensors are smaller components that have restricted battery capacity and they have no energy store capacity and have is usually utilised at once. In turn, the adoption of the low-energy routing strategy would result in the long sensor life cycle and improved network efficiency. Routing in the ad hoc sensor networks is an active research area at this point^14^. Ad-hoc routing has also greatly developed since the development of highly dynamic protocols such as DSDV^15^.

A number of strategies have been proposed in the current literature on DSR, and AODV, whereby one of them is outlined in^6^.

The Self-learning ad-hoc routing protocol is one more interesting approach because it proposes employing the Self-learning ad-hoc routing protocol combined with AODV to improve efficiency and integrate the route caching functions of DSR. Moreover, this paper presents a modified AODV Routing Protocol (EARP) which is based on AODV^16^. This study is mainly aimed at addressing the problem of frequent route expiration as well as to suggest a measure of the statistical estimate of the duration of route validity. Such criteria are quite effective in eliminating the number of route requests and, therefore, energy efficiency increases.

Energy preservation is a major worry due to the fact that ad hoc networks typically rely on battery power. Even the node acts as a router and relays messages to further networks, its battery depletes, even if it doesn’t have any messages of its own to transmit. In contrast to the Components such as microprocessors and communication, where processing capabilities and transfer rates have consistently improved (approximately average on every 18 months), battery technology has remain essentially unchanged for several decades^17^. The lifespan of the battery determines the duration an ad hoc network can remain operational. Consequently, research on energy-saving techniques for adhoc networks has experienced a significant surge over the past few years^18^.

Energy preservation is a topic of investigation across all levels of the traditional protocol stack, spanning from the broadcast layer to the transport layer and application layer. Substantial energy savings have been realized at various layers, including the physical layer, data connection layer, and network layer. In the radio layer, there are widespread practices such as low-power electronics, energy-efficient transmission methods, and the use of low signal power levels. Application of minimal level of transmitting voltage is also used in ensuring that connectivity and at the same time power efficiency and minimization of interference are assured.

Medium access control methods aimed at conserving power at the designed data layer are to manage the transition of nodes between listening and sleeping modes. It’s important to highlight that mobile power-saving techniques involve a close interconnection between different protocol layers. For instance, determining the minimum broadcast energy level may occasionally depend on GPS-enabled routing protocols for obtaining the geographic coordinates of nodes. Additionally, the structure of routing data exchange at the network layer plays a role in shaping the implementation of the sleeping state within the protocol stack^19^.

To address the need for enhanced energy efficiency at the network layer, Energy-Aware Routing (EAR) introduced protocols. The energy used by nodes for receiving and transmitting packets is commonly known as the energy consumption of the connectivity subsystem is considered. Energy requirements for data investigation and additional tasks are not factored in, as the computational energy consumption varies depending on the specific tasks of each node and is usually insignificant when compared to the power requirements of the module of transceiver. The initial objective of EAR was to identify the most efficient path to minimize the overall energy consumption of the network. The primary idea is to reduce the average power consumption per packet (as it travels through the network).per unit flow) or the network. One important disadvantage of this strategy, like with minimum-hop routing, is that nodes’ energy usage would vary greatly^20^. The nodes on the lowest energy paths will swiftly deplete, while the rest of the nodes will remain intact. As a result, some nodes die prematurely.

Modern wireless sensor and Internet of Things networks have been optimized and made energy aware, significantly boosting the performance and sustainability of such networks. The distributed parallel particle swarm optimization has been used to minimize the RFID reader collision and enhance the efficiency of large scale identification^21^. Such communication paradigms as low-power listening concurrently and those aiming at energy efficiency minimize the duty-cycling overhead and prolong network lifetime^22^. Offloading computation strategies have been computed based on learning and applied in vehicular networks to increase processing efficiency at dynamic networks^23^, and automotive radar optimization in spectrally busy networks increases communication reliability^24^. There is also the use of Bayesian optimization to better understand the adaptability to autonomous system in uncertain terrains^25^. The fundamental deep reinforcement learning methods also reduce consumption of energy when utilizing wirelessly powered IoT sensing systems^26^. Innovative transformer-based systems are used to overcome the temporal bias of IoT data analytics^27^, and Multipath adaptive routing plans are used to improve the energy efficiency in LEO-based industrial IoT systems^28^. More evidence of the combination of intelligent optimization with sustainable network functioning is given by quantum-enhanced heuristic optimization and resource-efficient environmental system design^29,30^.

Recent research has also covered energy-saving routing and clustering approaches to WSNs. For instance, optimal gateway placement and clustering approaches have been proposed to improve intra-cluster communication efficiency^31^. The stochastic optimization-based mobile sink-based routing has also been shown to have better energy balancing and data collection efficiencies^32^. Hybrid clustering and rendezvous-based data collection models have been proposed in order to provide higher scalability and communication overhead minimization in multi-sink WSN environments^33^. Moreover, there are delay conscious routing schemes like link-score-based routing that enhance quality of service on time sensitive applications^34^. The new developments also incorporate energy efficiency in cognitive radio sensor networks and adaptive networking schemes like self-healing schemes in software-defined networks that are more robust and spectrum efficient^35,36^.

Although much research has been done in energy-conscious routing and protocols based on Ant Colony Optimization, current strategies like ACA and EAACA have limitations in balancing the use of energy as well as efficient utilization of space in routing. The reason is that most of the existing techniques either concentrate on the intensity of pheromones or remaining energy without taking into account its combined effect together with the proximity of nodes. This gives rise to disproportionate energy loss and decreased network lifetime, particularly in dense and dynamic WSN set-ups. The main contributions of this work are as follows:

- A novel Lifetime-Aware Ant Colony Optimization-based Routing Algorithm (LTAWSN) is proposed.
- Combination of the dual energy metrics to enhance energy balancing with respect to nodes.
- Use of spatial proximity in routing decisions in order to minimize hop count.
- Development of a probabilistic routing model combining pheromone, energy, and distance factors.
- Extensive simulation-based comparison with ACLR, ACA, and EAACA protocols.

## Energy aware routing protocol

### Routing metric for the maximum system lifetime

Efficient routing techniques designed for power conservation operate within the network layer to select the most optimal route to reduce total energy consumption or increase system longevity. Instead of using the simple hop count measure, the shortest path techniques are coupled alongside other carefully built power-aware cost measures. It has been proposed to use power-aware measures to determine routes with diverse goals. Equation (1) is a metric for maximizing the lifetime of all network nodes

1 $$\:{c}_{j}={\sum\:}_{i=1}^{k-1}{f}_{i}\left({x}_{i}\right)\:$$

In the context where *cj*​ denotes the cost associated with packet transmitting *j* from node $\:{n}_{1}to{n}_{k}$through an intermediate node n2ynk 1, *xi*​ stands for the cumulative energy consumption of node *i* up to this point, and *fi*​(*xi*​) signifies the cost or importance assigned to node *i* in terms of its reluctance to forward packets (notably denoted as *fi*​ due to its representation in Eq. (2)).

2 $$\:{f}_{i}\left({x}_{i}\right)=\frac{1}{{E}_{i}-{x}_{i}}$$

Where *fi*​(*xi*​) signifies the present utility or desirability of utilizing node *i*, where *xi*​ reflects the cumulative energy expended by node *i* up to the current time (notably, *xi*​ varies over time). *Ei*​ denotes the initial energy level of node *i* when the system was initially established. Consequently, *fi*​ represents the inverse of the remaining energy in node *i*’s perspective. As a node’s energy diminishes, the desirability of utilizing that node increases.

The function in Eq. (2) represents the inverse of the remaining energy of node i. The less the residual energy, the higher the cost of the choice of the node. This is a deterrent of path routing across nodes with low energy and ensures equal distribution of energy in the network.

### EAR performance

**Fig. 1:**
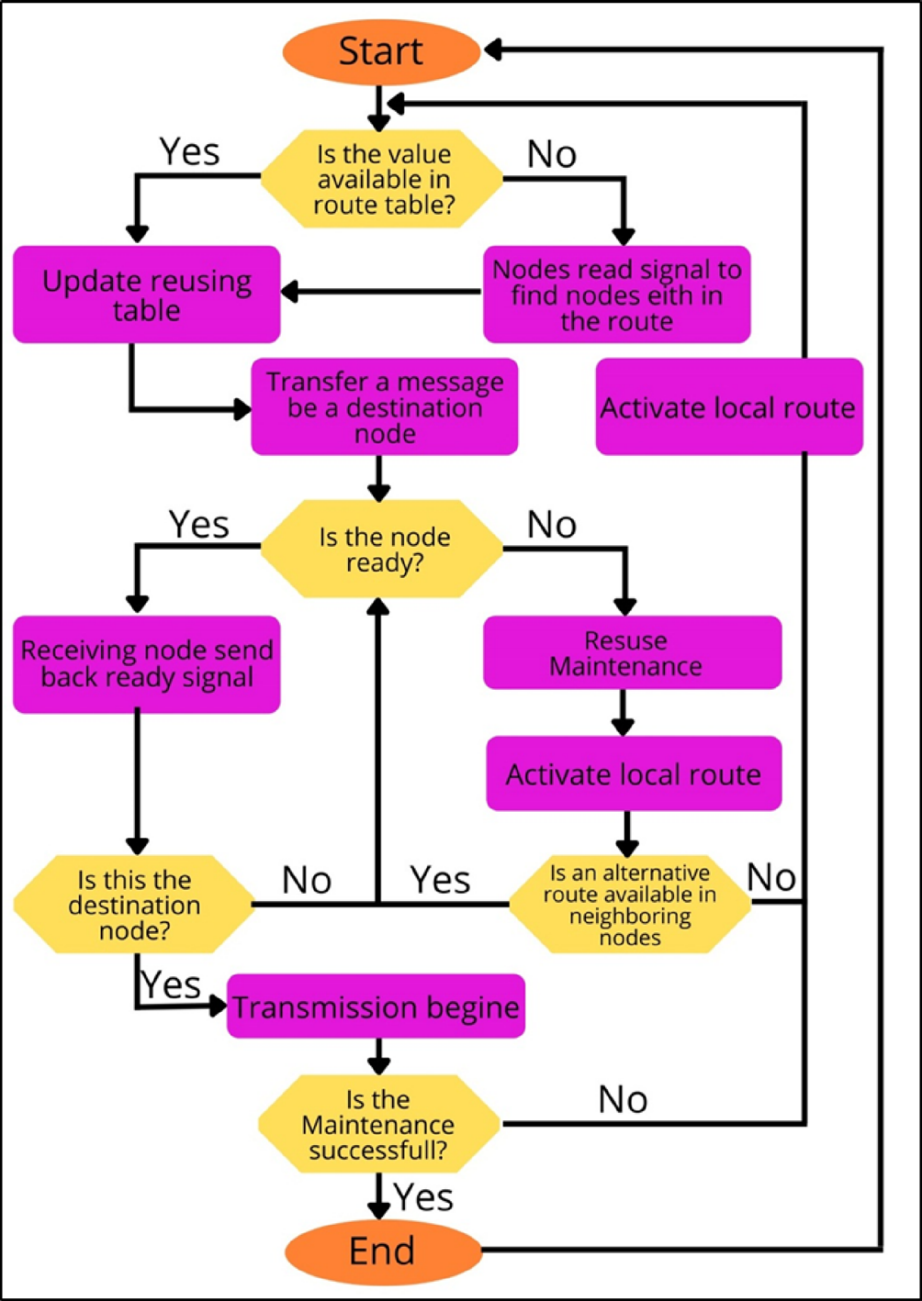
Flowchart for the AODV routing protocol.

Where more than one route exists between a source to a destination, EAR uses a tactic of alternating between different routes making a move to allocate traffic load in a manner that best avoids waste of the remaining energy. Resultantly, this has a life span of a system that is increased beyond the traditional methods of routing. In order to determine its effectiveness, we were simulating it and comparing it with traditional ad hoc routing methods. In carrying out these simulations, we have used the GloMoSim package which is a modelling framework that is scalable to wireless communication networks. CBR traffic is used, with each packet being 512 bytes long. The data transmission rate between each source and destination (referred to as a session) is randomly determined using an empirical function, with an average rate of one message per second. Each set of simulations involves varying numbers of networks and sessions. To ensure a fair evaluation of alternative routing protocols, we standardized the configuration of each model. In our model, we specifically assess the energy consumption associated with the transmission subsystem, which aligns with the approach taken in most EAR protocols. Before deploying the network, each node was allocated a fixed amount of energy or battery backup, equivalent to 20,000 energy units. We consider the cost incurred by a node for transmitting and receiving packets as a combination of two components: There is a constant cost linked to channel allocation, and an added expense that increases proportionally with the packet size. You can see the flowchart of the AODV routing protocol in Fig. 1.

### Energy-aware route discovery

Energy-aware routing methods calculate connection and route costs by considering energy-conscious parameters. Energy-aware routing protocols employ the min-max optimal routing approach. In traditional route discovery systems, the process begins with the transmission of a request, intended to be received by nearby nodes that subsequently repetition of the message. These repetition packets are audible to all nodes. Nodes that have previously relayed the request disregard it. Ultimately, the node at destination responds to the received request, but the most energy-efficient route is not established during the entire process. Consequently, the protocols need to modify their methods for discovering paths to give priority to energy efficiency.

### EARM

Only when links were broken owing to the distribution of route error packets was route maintenance conducted. There’s no way to tell if node mobility is causing a change in network reliability or energy costs. The Minimizing the energy wastage is essential, by monitoring changes in the energy prices of the links on a regular basis. To accommodate node mobility, adjustments in transmission power are necessary. The power should decrease as nodes approach each other to conserve energy and increase as nodes move farther apart to sustain the connection. Consequently, these fluctuations in energy expenses need to be conveyed to the source node, enabling it to opt for alternative, more cost-effective routes when necessary. As a result, an energy-efficient routing protocol should include a system for monitoring fluctuations in link energy costs.

### Design parameter

The proposed algorithm is created using the parameters listed below. In addition, the same factors are utilized to compare and validate the two distinct setups.

#### PDF

The PDF metric holds significant importance as it characterizes the loss rate or The highest achievable network throughput. Various factors include collision of packets occurring at the layer of 802.11, network segmentation, routing loop occurrences, and interface queues drops, can contribute to packets failing to reach their intended destinations. A high PDF score indicates that the protocol is performing effectively since it signifies that the majority of packets successfully reach the upper layers of the network. Equation (3) is used to get the PDF.

3 $$\:\:PDF\:=\frac{NumberofpacketsRecitedbytheDestination}{NumberofpacketssentbySource}$$

#### ECSDD

ECSDD stands as the representing entity for this metric. It quantifies the ratio of the total network energy consumption to the total number of packets successfully transmitted to the destination node. In this, the power usage of the network should include all the energy used not including the MAC layer controls as in Eq. (4).

4 $$\:ECSDD=\frac{{\sum\:}_{k=1}^{N}\left({E}_{ik}-{E}_{rk}\right)\:\:}{TotalEquationNumberofpacketsreceived}$$

Here, *Eik*​represents the node *k* initial energy, *Erk*​ signifies node (*k)* energy level at conclusion model, and *N* denotes the total node count within the network. A lower ECSDD value would imply that a large percentage of packets have been transmitted using less energy consumption, which would mean that the protocol has become more energy efficient.

#### VRBE in Joules

It is one of the basic measuring tools of energy balance that can be used to extend the lifetime of your network. The result obtained in the calculations indicates the routing system has employed a plentiful number of sensor nodes. As a protocol-agnostic measure, it is of paramount significance as a performance measure. As such, a value of zero or close to zero should be the ideal value of this measure. A smaller VRBE implies that all network nodes are treated equally in terms of importance, without any single node being penalized more than the others. You can find it represented in Eq. (5).

5 $$\:{V}_{RBE}=\frac{{\sum\:}_{k-1}^{N}{\left({E}_{zk}-\mu\:\right)}^{2}\:\:}{N};Where\mu\:=\frac{{\sum\:}_{k-1}^{N}\:{E}_{rk}}{N}$$

#### N)

It stands as one of the crucial metrics to evaluate the energy efficiency of routing methods in the context of network partitioning. In a Ad Hoc wireless network, particularly one with nodes densely distributed, the network’s complete collapse is seldom a consequence of the first node’s failure. Rather than experiencing an immediate collapse, the network undergoes fragmentation as the count of non-functional nodes grows. indeed in the event of network segmentation, end-to-end communication remains feasible if at least one pair of proximate nodes relics operational, enabling them to communicate with each other and sustain the system’s functionality. Consequently, NL can be described in the following manners: (1) This can be defined as the duration required for K percent of a network’s nodes to become non-operational, (2) In the context of a specific data flow, the network’s lifespan can be described as the duration until the battery depletes.

### Proposed methodology

#### Routing algorithm based on ACO proposed (LTAWSN)

In this section, we’ll explain how the LTAWSN method works. First, a WSN routing method depending on conventional ant colony optimization is described. The EAACA algorithm is then introduced. Lastly, the LTAWSN routing method is introduced, which aims to enhance energy usage while also ranging the network lifetime.

**Figure:**
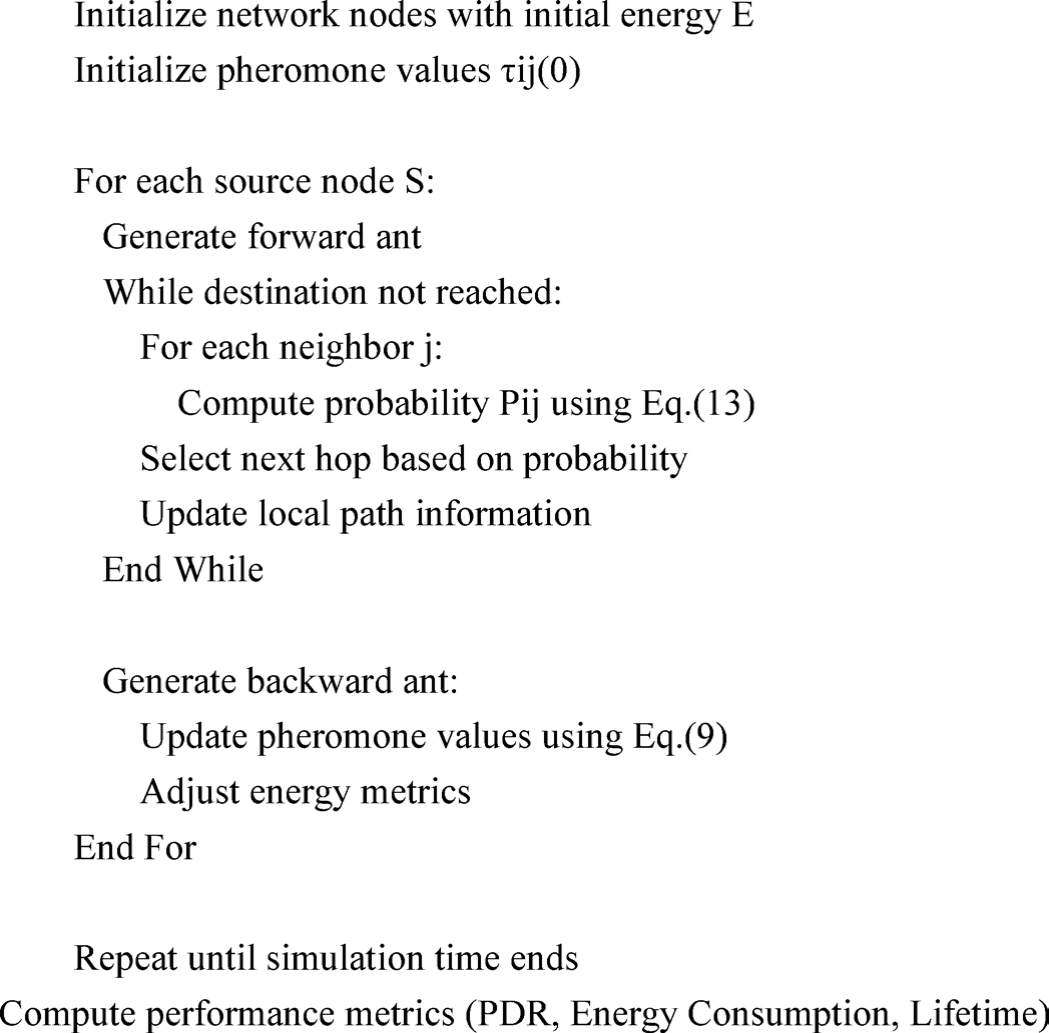
Pseudocode of proposed LTAWSN algorithm.

The proposed LTAWSN algorithm has two key stages: forward ant exploration, and backward ant reinforcement. At the forward stage, ants are probabilistic in choosing the next hop according to Eq. (13) that takes into account the concentration of pheromones, remaining energy and proximity to the nodes. This makes sure that preference is given to nodes with greater energy, and those that are nearer to the destination. After reaching the destination, the ants go back in the same direction, and rewrite the pheromone levels to make efficient routes stronger. Moreover, the dynamically updated energy metrics are used to indicate node energy consumption. This two-stage system facilitates dynamic routing and on-the-fly optimization of routes in a network.

#### Basic WSN routing based on ACO (ACA)

A weighted undirected connection graph G can be used to depict WSN (V, E). Where V is the number of sensor nodes and E is the number of connections between them. Any node in the WSN area has a group of individuals who are located within the node’s wireless transmission coverage. The distance between 2 nodes in the WSN region is calculated using the Euclidean distance. The Euclidean length between I and j is computed using Eq. (6).

6 $$\:{d}_{ij}=\sqrt{{\left({x}_{i}-{x}_{j}\right)}^{2}+{\left({y}_{i}-{y}_{j}\right)}^{2}}\:;\:i=\left({x}_{i},{y}_{i}\right);\:j=\left({x}_{j},{y}_{j}\right)$$

Ant-based routing techniques detect and optimise routes between nodes by using ant-like control messages. These ants are query messages that try to find all possible connections between the origin and target nodes. The approach assumes that each sensor network has only one detector destination and uses 2 ants types: forward ants, originating from the source node and journeying toward the destination node, uncovering new paths and gathering data, and reverse ants, originating from the destination node, moving backward and updating data in each sensor node as they advance. They achieve this by depositing pheromones along a pre-defined path. Pheromone levels regulate the movement of ants traveling from one network to another along a multi-hop route enroute to a destination node. In a traditional ACO-based routing protocol, the likelihood of an ant moving from a specific node I to another node j is defined by Eq. (7).

7 $$\:{P}_{ij}^{k}\left(t\right)=\frac{{\left[{\psi\:}_{ij}\left(t\right)\right]}^{\alpha\:}\times\:{\left[{\epsilon\:}_{jj}\left(t\right)\right]}^{\beta\:}}{{\sum\:}_{s,C\left(s\right)}^{}\:\:{\left[{\psi\:}_{il}\left(t\right)\right]}^{\alpha\:}\times\:{\left[{\epsilon\:}_{il}\left(t\right)\right]}^{\beta\:}}.$$

$\:{P}_{ij}^{k}\left(t\right)$ has been the probability of a packet being transferred from node i to node j for ant k in time t, $\:{\left[{\psi\:}_{ij}\left(t\right)\right]}^{\alpha\:}$ signifies The pheromone concentration gathered on path segments i and j by ants over time,$\:{\left[{\epsilon\:}_{jj}\left(t\right)\right]}^{\beta\:}$ corresponds to the exploration data for that route segment, and a and b are two constant values associated with the algorithm. The recommended location function for the classical routing algorithm is defined in Eq. (8).

8 $$\:{E}_{ij}=\frac{1}{{C}_{ij}}$$

where$\:{c}_{ij}$ denotes the distance between nodes *\:I* and *\:j*in Euclidean space.When the ant discovers the target node, it establishes a path between the source and target nodes. Subsequently, the target node dispatches a response packet, often referred to as a “backward ant.” The backwards ant releases pheromone while returning to the transmitting node along the backward direction. At the end of each searching time, the pheromone $\:{\psi\:}_{ij}$would be updated as per Eq. (9)

9 $$\:{\psi\:}_{ij}(t+1)=(1-\rho\:)\times\:{\psi\:}_{ij}\left(t\right)+\varDelta\:{\psi\:}_{ij}\left(t\right)$$

#### EAACA for routing of WSN

The residual energy of the node is taken into account in EAACA while computing the packet transmission likelihood to the next hop neighbor. Ants are discharged from a origin address and hop from one node to the next on their way to the target node. As per a probabilistic choice rule, ant k in node I choose the next node j to go to (refer Eq. (10)).

10 $$\:{P}_{ij}^{k}\left(t\right)=\frac{{\left[{\psi\:}_{ij}\left(t\right)\right]}^{\alpha\:}\times\:{\left[{\mu\:}_{ij}\left(t\right)\right]}^{\beta\:}}{{\sum\:}_{s\in\:C\left(i\right)}\:{\left[{\psi\:}_{il}\left(t\right)\right]}^{\alpha\:}\times\:{\left[{\mu\:}_{ij}\left(t\right)\right]}^{\beta\:}}.$$

Transfer packet likelihood and pheromone measure are represented by $\:{P}_{ij}^{k}\left(t\right)$, and $\:{\left[{\psi\:}_{ij}\left(t\right)\right]}^{\alpha\:}$, respectively. EAACA suggested the energy function, which is formally defined in Eq. (11).

11 $$\:{\mu\:}_{ij}=\frac{1}{E-{e}_{j}\left(t\right)}$$

Where E denotes the node’s starting energy and e_j_(t) is the node’s actual energy at time t. The pheromone $\:{\psi\:}_{ij}(t+1)$ in EAACA will be updated at the end of each searching time in Eq. (12).

12 $$\:{\psi\:}_{ij}(t+1)=(1-\rho\:)\times\:{\psi\:}_{ij}\left(t\right)+\frac{\varDelta\:{\psi\:}_{ij}\left(t\right)}{\omega\:\cdot\:{\:hop\:}_{coset\:{k}_{k}}}$$

#### An Ant Colony Optimization-based routing system for Wireless Sensor Networks that extends network lifetime (LTAWSN)

Researchers provide an ant colony-based routing strategy with unique competence function variables in this section, with the goal of reducing network node energy usage while also producing a more balanced distribution across nodes and extending the network’s lifetime. The fundamental prerequisites for optimal performance in a Wireless Sensor Network (WSN) are minimal power consumption and extended operational lifespan. As a result, energy consumption must be factored into route design.

Our designs aim to establish a system that evenly distributes total energy absorption across all network nodes. To increase routing efficiency and energy utilisation, a compromise between route hops and power consumption is required.

In the LTAWSN technique, The list of potential neighbors for each node comprises nodes located within the node’s wireless communication range and are also closer to the target node than the current node. Because energy is a critical aspect in the competency activity, we use two energy measures in this function, each with its own specification. On the other hand, we incorporate a spatial variable in the LTAWSN method’s probability distribution function based on the notion that the node in the neighbour list of candidates for each current node that is closest to the target node has a better chance of reaching the destination node with fewer hops.This parameter takes into account the distance between candidate list nodes and the destination node, with nodes closer to the target node having a higher probability of being chosen. This probabilistic decision criterion is applied to the probability distribution function of the LTAWSN method for choosing the next-hop node in present travel (refer Eq. (13)).

Equation (13) combines pheromone strength, remaining energy and geographical proximity in to one probabilistic system. The parameters of control (a, b, c, d) control the effect of the factors of energy and distance. This expression guarantees that the routing decisions are made based on the preservation of energy as well as hop efficiency consequently prolonging network lifetime.

13 $$\:{P}_{ij}^{k}\left(t\right)=\frac{{\left[{\psi\:}_{ij}\left(t\right)\right]}^{\alpha\:}\times\:{\left[{\eta\:}_{ij}\left(t\right)\right]}^{\beta\:}\times\:{\left[{\eta\:}_{ij}^{{\prime\:}}\left(t\right)\right]}^{\gamma\:}\times\:{\left[{\epsilon\:}_{ij}\left(t\right)\right]}^{b}}{{\sum\:}_{{s}_{i}\in\:C\left(i\right)}\:{\left[{\psi\:}_{ai}\left(t\right)\right]}^{\alpha\:}\times\:{\left[{\eta\:}_{il}\left(t\right)\right]}^{\beta\:}\times\:{\left[{\eta\:}_{ij}^{{\prime\:}}\left(t\right)\right]}^{\beta\:}\times\:{\left[{\epsilon\:}_{il}\left(t\right)\right]}^{\delta\:}}$$

$\:{P}_{ij}^{k}\left(t\right)$, $\:{\left[{\psi\:}_{ij}\left(t\right)\right]}^{\alpha\:}$ are the probability of transfer packets and the pheromone metric, etc. a; b; c; d is the control factors, and $\:{\eta\:}_{ij}\left(t\right)$represents the first power metric with the following description (refer Eq. (14))

14 $$\:{\eta\:}_{ij}\left(t\right)=\frac{{e}_{j}\left(t\right)}{{\sum\:}_{{s}_{i}\in\:C\left(i\right)}\:{e}_{l}\left(t\right)}$$

**Figure:**
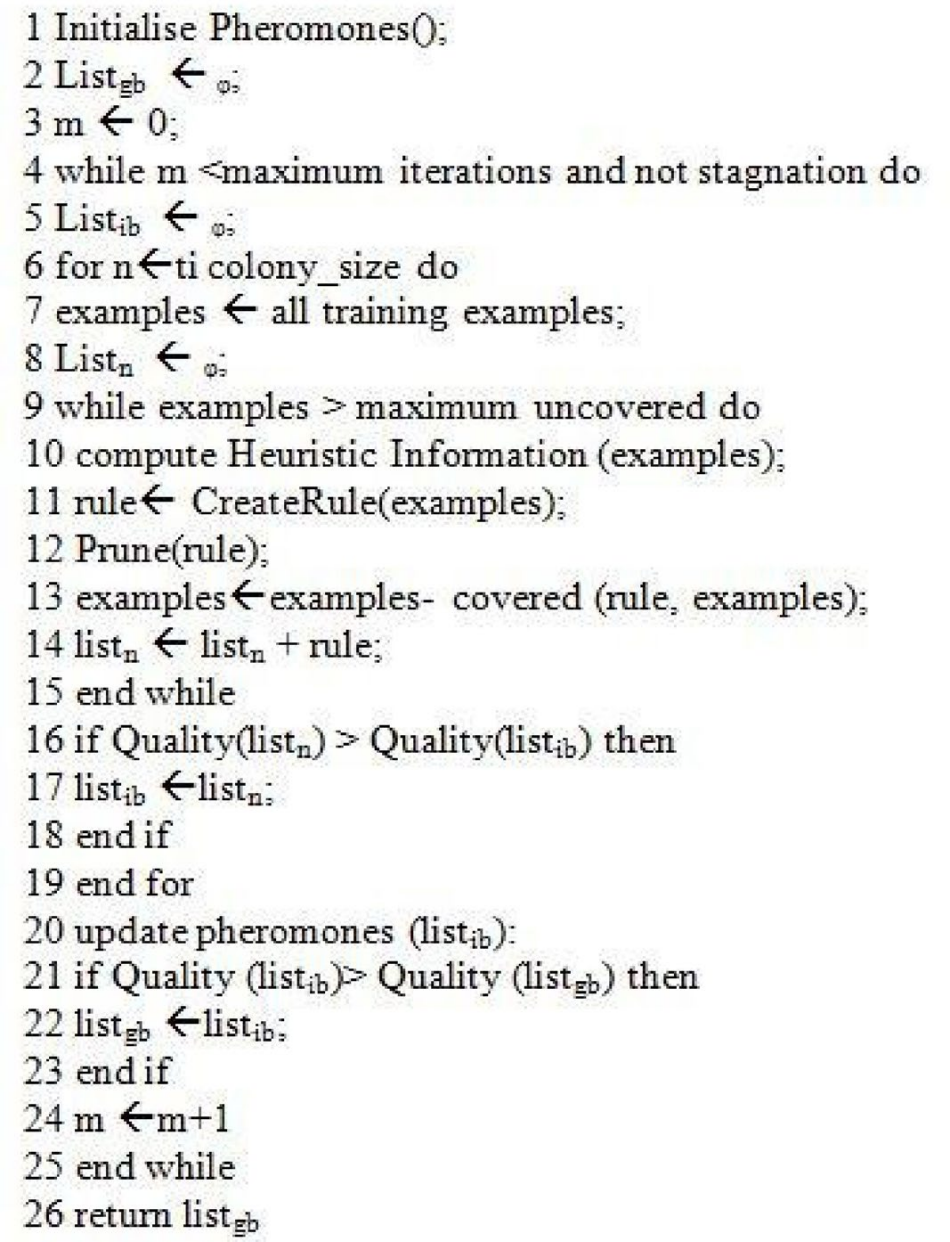
Ant Colony Optimization Algorithm.

The primary novelty of this work lies in integrating dual energy metrics with spatial awareness into the ant colony optimization framework. Unlike conventional ACA and EAACA approaches that consider either pheromone intensity or residual energy independently, the proposed LTAWSN algorithm introduces a combined probabilistic decision mechanism that balances energy consumption and hop efficiency. Additionally, the algorithm dynamically adjusts routing probabilities based on residual energy distribution, resulting in improved load balancing and extended network lifetime.

#### Computational complexity

The LTAWSN algorithm proposed is mainly dependent on the number of nodes and the frequency of ant generation, which is the most important factor in the computational complexity of the algorithm. In comparison to traditional ACA, the added energy and spatial parameters have a minor effect as they add to the computation overhead. Nevertheless, this is a small increase and is warranted by a large increase in energy efficiency and network lifetime.

## Experimental results

### Simulation environment

The suggested approach was tested with the help of the NS2 simulator that contains the wireless scenarios in its configuration. In order to facilitate the model to the proposed approach, adjustments were done to the The integration of energy related feedback by installing the No Ad-hoc Routing protocol patch on the “mobile node.cc” and “mobile node.h” files in ns-2.

The test environment The test environment is a 50 node network which is confined within a rectangle that is 1800 × 840 square meters. At the very beginning, every node is supplied with 1000.0 J of power. The TwoRayGround model is used to make sure it is realistic. The requirements for each simulation model depend on the need of the model. The scenario analysis is based on the variation of the amount of nodes. Continuous Bit rate (CBR) is applied in creating traffic and every packet measures 512 bytes. Here, The network design and the number of nodes are fixed with the only known giveaway of the number of origin-target pairs in the network. The simulation helps to analyze and test methods especially in large-scale systems. The list of simulation variables is provided in Table 1.

In order to bring out experimental reproducibility, all simulations were carried out with NS-2 with the same configuration parameters among comparative algorithms. The transmission power (Tx) and reception power (Rx) were set at 1.0 W with each node starting off with 1000 Joules of energy. The length of the queue was restricted to 100 packets to prevent bias in buffer overflow. The duration of the simulation was optimized based on the density of the nodes so that steady-state performance assessment was sustained. Five separate performances of each scenario with various random seeds were done and reported results are mean values. This rational arrangement will ensure an equal comparison between ACLR, ACA, EAACA, and the suggested LTAWSN algorithms.

**Table 1:** Simulation configuration.

Parameter	Value
Type of antenna	Omni antenna
Type of channel	Wireless channel
Interface queue type	Drop tail/Pri queue
MAC type	802_11
Maximum packet in the queue	100
Type of network interface	Phy/wireless phy
Node count	25, 30, 40, 50
Size of packets	1060 bytes
Wireless propagation model	Two ray ground
Protocols for routing	Energy efficient power aware routing protocol
Rx power	1.00 W
Node initial energy	1000.0 Joules
Topographical area	1800 × 840 sq.m
Total simulation time	As per requirement
Traffic model	FTP
Tx Power	1.00 W

### Results and discussion

The node system was first configured in the following manner.

#### SYSTEM

**Fig. 2:**
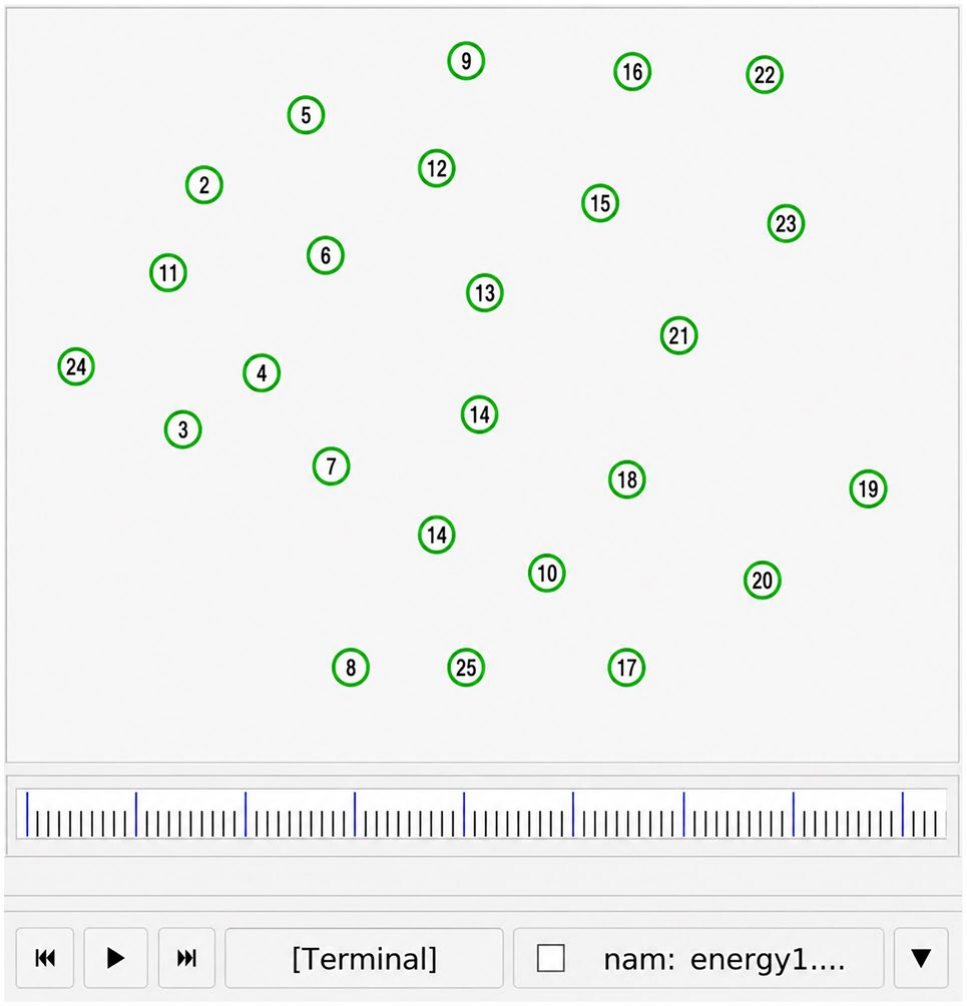
Modeling a network of 25 nodes.

The first setup has 25 nodes, with the established pause time being 25 milliseconds., the size of the packet list is 100, and the size of single packets is 1060 bytes. Running the model leads to production of a graph which shows average latency, Packet Delivery Ratio (PDR), throughput, and energy remaining. Figure 2 provides a general description of the position of the nodes in setup-1 having a total count of 25 nodes.

##### E-to-E delay on average vs. pause time

**Fig. 3:**
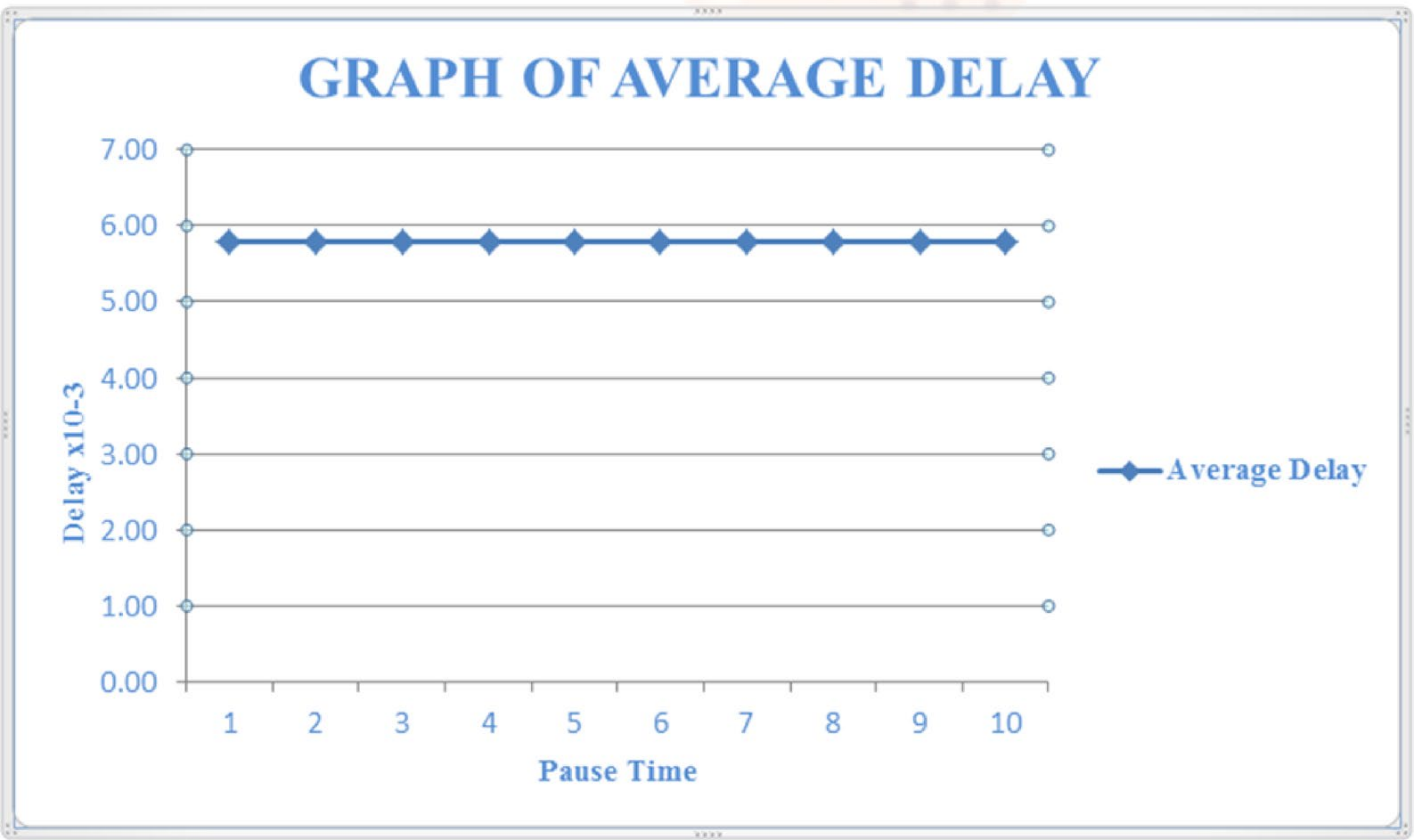
Average End-to-End Delay vs. Pause TimeGraph.

Figure 3 illustrates the graph depicting the relationship between average delay and pause time. The “Average Delay from Source to Destination” refers to the mean duration for packets to traverse from one end of the network to the opposite end. Since all other flows exhibit similar delays, this graph pertains solely to the first flow. It’s important to highlight that, after the route is established, the delay remains consistent as evidenced by the graph above. Figure 3 demonstrates that the latency has remained consistent and unchanged.

##### Pause time vs. PDR

**Fig. 4:**
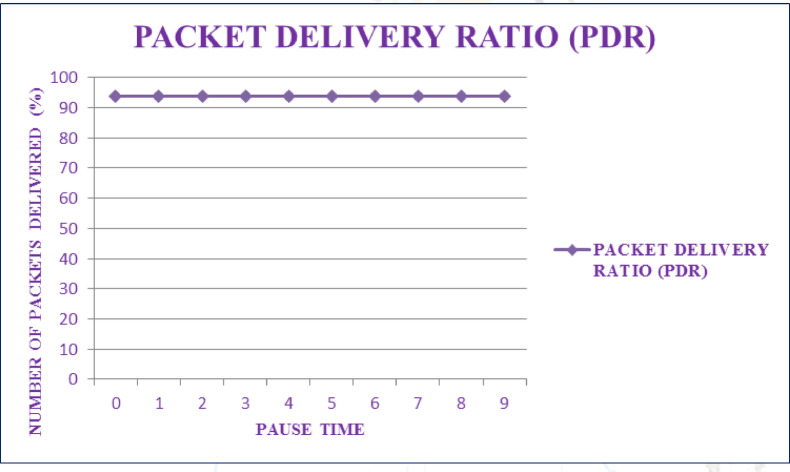
PDRVs Pause time Graph.

PDR chart for a 25-node simulated scenario is shown in Fig. 4. A 25-node design has a PDR of 94%. Figure 4 shows that PDR is substantial.

**Fig. 5:**
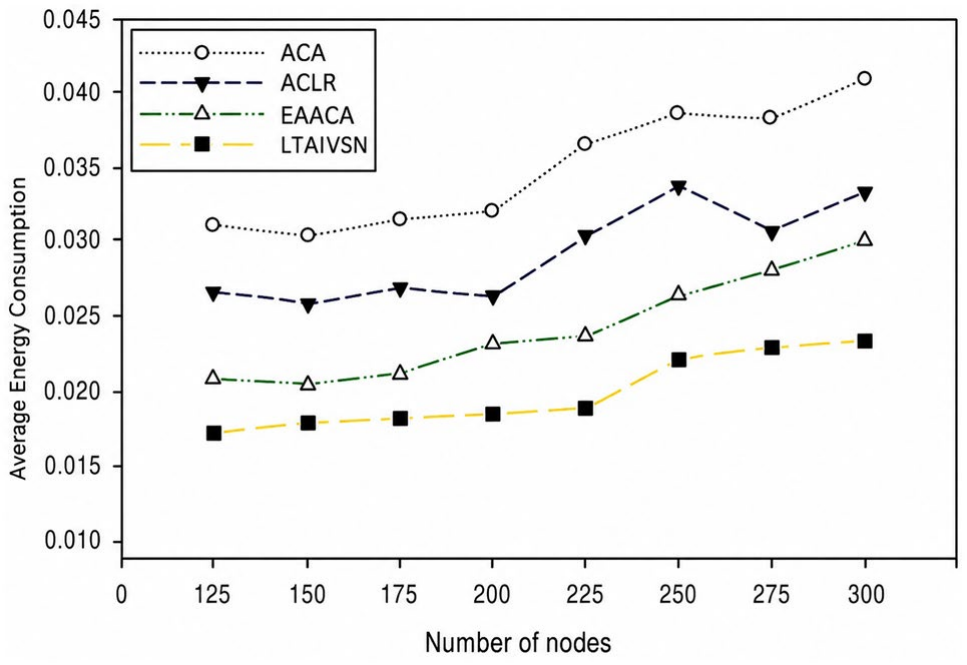
The average energy usage for a network of 100 9 100 m^2^.

**Fig. 6:**
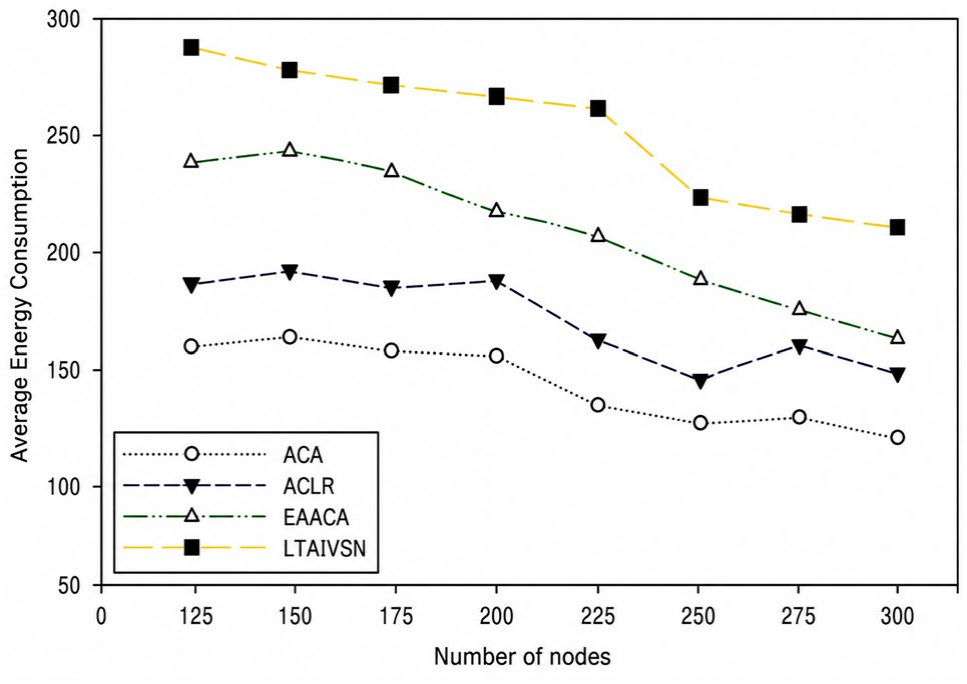
Lifespan of the network with the size of 100 9 100 m^2^.

WSN nodes are uniformly distributed in a certain region for all algorithms. The factors employed in stimulation are listed in Table 1. The wireless interaction radius of the sensor is R, the pheromone evaporation rate is q, the initial secretion layer for each pair of adjacent nodes iswijð0Þ, the node’s initial energy is E, Et is the power usage per bit broadcasted is Et, and Er is the power consumption per bit obtained in this table is Er. Analyses of simulated findings are based on the assumption that m sensor nodes are randomly positioned in the simulation study’s monitoring zone, with m ranging from 125 to 300.The primary criterion for evaluating routing methods is average energy usage. The result of these separate experiments is energy usage. The methods that each runs on networks with varied nodes are depicted in Fig. 5. The longevity of these networks is depicted in Fig. 6. To demonstrate the validity of the proposed method, two simulations with different network lengths are shown.

**Fig. 7:**
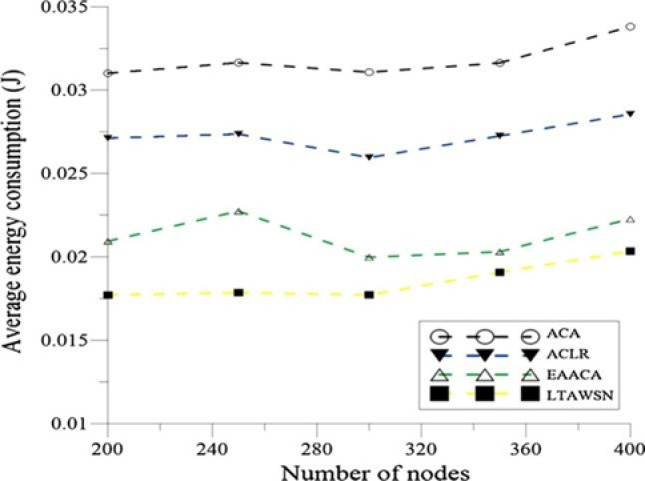
The average energy usage for a network of 200 9 20 m^2^.

**Fig. 8:**
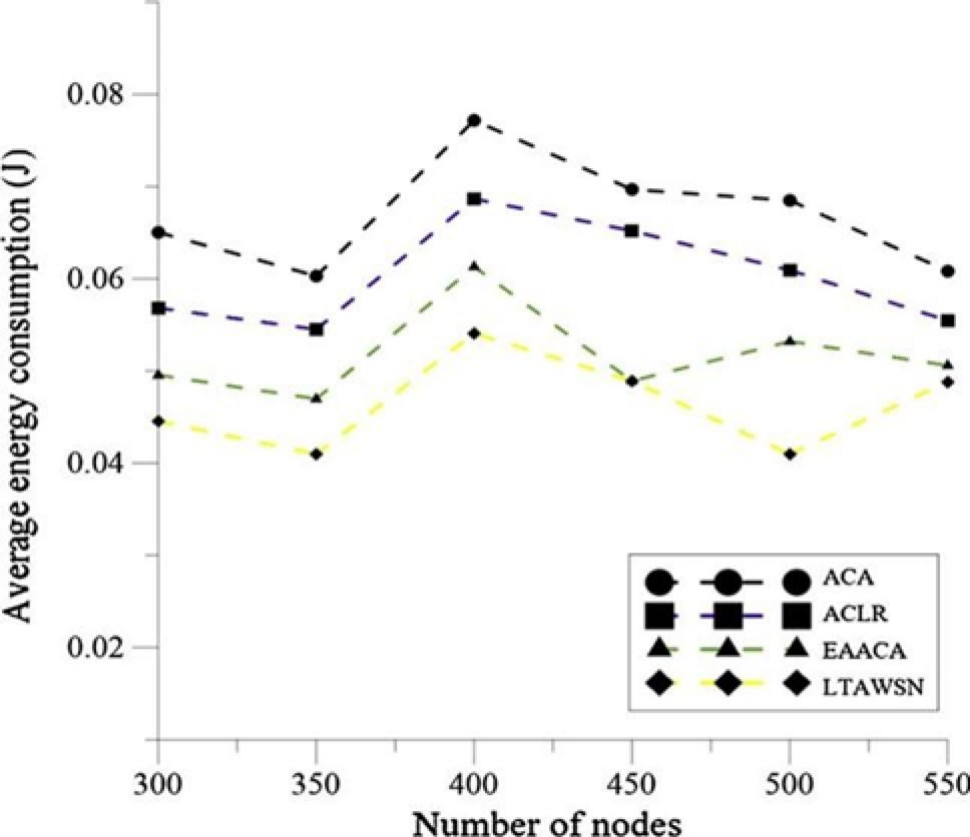
The average energy usage for a network of 500 9 500 m^2^.

Figure 7 shows the outcome of energy usage for a network with a different architecture. Figure 8 also shows the energy usage for various network sizes and topologies. The number of nodes evaluated in Fig. 4 ranges from 330 to 550. The modeling results show that the proposed strategy reduces average energy use. According to simulation findings, the proposed technique provides the best performance in all tested architectures. The energy requirement of nodes in the suggested algorithm is less than in the ACLR, ACA, and EAACA algorithms, as can be seen. The network’s energy usage is reduced to lengthen the network’s lifespan. As a result, the suggested approach extends the network’s life and improves its reliability. Tables 2 and 3 indicate the parameters utilized in these 2 simulations, etc. The number of sensor nodes in this model ranged from 200 to 400.

The proposed LTAWSN algorithm was compared against mainstream energy-aware routing approaches, including ACLR, ACA, and EAACA. These comparative outcomes signify that the LTAWSN operates at low average energy consumption because it has the duality of energy metrics in the decision mechanism. LTAWSN also uses the residual energy awareness as compared to ACA, which mostly uses the strength of the pheromones, and as such, avoids a situation where a critical node is drained off prematurely. The proposed algorithm introduced as an improvement over the EAACA, where the residual energy is taken into account, although nothing is done to optimize the space, will lead to fewer hops and less overhead on the transmission.

Thus, LTAWSN provides better Packet Delivery Ratio (PDR) and even higher network lifetime. Nonetheless, the suggested algorithm comes with a few more extra complexities in terms of probability calculations. This loss is tolerable when one takes into consideration the major improvement in terms of energy efficiency and stability.

The LTAWSN scheme suggested could be used especially in energy-limited Internet of Things (IoT) systems, smart agricultural systems, battlefield databases, disaster recovery systems, and industrial surveillance. Balanced routing can be used in large-scale IoT deployments, where nodes are required to operate within stringent energy constraints, in order to lower operational lifetime. Moreover, stable energy-constrained routing is a more reliable performance in delay-sensitive and mission-critical systems (i.e., industrial automation and intelligent transportation systems) to prevent interruptions in the information flow. LTAWSN has also been flexible to the heterogeneous WSNs architecture in which different node capability and energy reserves differ because of the use of probabilistic decision mechanism. Hence, the suggested scheme has a good potential of being harnessed into the next-generation wireless communication solutions that demand sustainable and resilient networking systems.

Figure 5 shows the comparison of average energy consumption in LTAWSN, ACA, EAACA and ACLR algorithms. It is found that LTAWSN has always lower energy consumption owing to its balanced routing and dual-energy conscious decision mechanism.

**Table 2:** Modeling results for a network with a size of 200 9 200m2.

Parameter	Values
Network size	2009200m^2^
R	40 m
Number of nodes	125–300
Ant number	20
*Q*	0.8
*wij*ð0Þ	0.01
E	1 J
Et	4.28lJ/bit
Er	2.36lJ/bit
MAClayerprotocol	IEEE802.11

**Table 3:** Modeling values for a network of 500 9 500 m^2^ in size.

Parameter	Values
Network size	5009500m^2^
R	50 m
Number of nodes	125–300
Antnumber	20
Q	0.8
*wij*ð0Þ	0.01
*E*	1 J
Et	4.28lJ/bit
Er	2.36lJ/bit
MAClayerprotocol	IEEE802.11

The comparison from Table 4 clearly indicates that the proposed LTAWSN algorithm outperforms existing routing protocols by integrating both energy-aware and spatial-aware decision parameters. Unlike ACA, which lacks energy awareness, and EAACA and ACLR, which partially consider energy metrics, LTAWSN incorporates dual energy factors along with node proximity. This combined approach enables more balanced energy consumption across nodes, reduces the likelihood of premature node failure, and significantly enhances overall network lifetime. Furthermore, the inclusion of spatial awareness minimizes hop count and routing overhead, resulting in improved load balancing and more efficient data transmission in Wireless Sensor Networks.

**Table 4:** Comparative analysis of routing algorithms.

Algorithm	Energy awareness	Spatial awareness	Load balancing	Network lifetime
ACA	No	No	Low	Low
EAACA	Yes	No	Medium	Medium
ACLR	Partial	No	Medium	Medium
LTAWSN	Yes (Dual)	Yes	High	High

The LTAWSN algorithm proposed in the suggested algorithm attains an average decrease of 18–25% in energy consumption as compared to EAACA and ACA algorithm. Equally, the ratio of delivering packets (PDR) is enhanced by an average of 6–10 per cent indicating an increased reliability of data transmissions. Moreover, the lifetime of the network is greatly increased, especially at different node densities, since the dual energy-aware routing mechanism provides a balance in the use of energy.

## Conclusion

The paper has suggested that Lifetime-Aware Ant Colony Optimization-based Routing Algorithm (LTAWSN) can be implemented to Electronic Web in terms of Wireless Sensor Networks on how to deal with energy imbalance between nodes and sacrificial & untimely death of nodes. The combination of the remaining energy, hopcount, and geographical proximity in to one probabilistic routing paradigm makes the proposed approach attain higher efficiency and evenly dispersed loads. Analysis of the simulation outcomes proves the fact that LTAWSN will consume less energy, have better packet delivery ratio, and will dramatically increase network lifetime when compared to ACLR, ACA, and EAACA algorithms.

Further studies can be conducted to enhance the proposed work to include mobility-aware routing mechanisms and hardware implementation of IoT in real time. Moreover, the adaptability of the proposed approach can be also improved by incorporating machine learning methods to tune the parameters adaptively and investigate security-aware routing policies.

## Data Availability

The datasets used and/or analysed during the current study available from the corresponding author on reasonable request.
